# ‘It's about collaboration’: a whole-systems approach to understanding and promoting movement in Suffolk

**DOI:** 10.1186/s12966-024-01688-2

**Published:** 2025-01-16

**Authors:** A. J. Brinkley, K. M. Cusimano, P. Freeman, R. Southall-Edwards, V. F. Gladwell

**Affiliations:** 1 Sport, Rehabilitation and Exercise Sciences, University of Essex, Essex, CO4 3SQ UK; 2 Institute of Health and Wellbeing, University of Suffolk, Suffolk, IP4 1QJ UK

**Keywords:** Exercise, Health, Intervention, Leverage points, Physical activity, Sport, Wellbeing

## Abstract

**Background:**

Population-levels of physical activity have remained stagnant for years. Previous approaches to modify behaviour have broadly neglected the importance of whole-systems approaches. Our research aimed to (i) understand, (ii) map, (iii) identify the leverage points, and (iv) develop solutions surrounding participation in physical activity across an English rural county.

**Methods:**

A systems-consortium of partners from regional and local government, charities, providers, deliverers, advocacy groups, and health and social care, and public health engaged in our research, which consisted of two-phases. Within Phase 1, we used secondary data, insight-work, a narrative review, participatory workshops, and interviews in a pluralistic style to map the system-representing physical activity. Phase 2 began with an initial analysis using markers from social network analysis and the Action Scales Model. This analysis informed a participatory workshop, to identify leverage points, and develop solutions for change within the county.

**Results:**

The systems-map is constructed from biological, financial, and psychological individual factors, interpersonal factors, systems partners, built, natural and social environmental factors, and policy and structural factors. Our initial analysis found 13 leverage points to review within our participatory workshop. When appraised by the group, (i) local governing policies, (ii) shared policies, strategies, vision, and working relationships, (iii) shared facilities (school, sport, community, recreation), and (iv) funding were deemed most important to change. Within group discussions, participants stressed the importance and challenges associated with shared working relationships, a collective vision, and strategy, the role of funding, and management of resources. Actions to leverage change included raising awareness with partners beyond the system, sharing policies, resources, insight, evidence, and capacity, and collaborating to co-produce a collective vision and strategy.

**Conclusions:**

Our findings highlight the importance and provide insight into the early phase of a whole-systems approach to promoting physical activity. Our whole-systems approach within Suffolk needs to consider methods to (i) grow and maintain the systems-consortium, (ii) create a sustainable means to map the system and identify leverage points within it, and (iii) monitor and evaluate change.

**Supplementary Information:**

The online version contains supplementary material available at 10.1186/s12966-024-01688-2.

## Background

Consistent with long-term trends, across the UK, and the globe [1], 36.9% of adults [2] residing in England are physically inactive (i.e., do not meet physical activity (PA) guidelines; > 150 min of moderate intensity PA per-week) [3, 4]. PA is a modifiable risk-factor for a multitude of non-communicable diseases [5], poor mental health and wellbeing [6, 7], reduced quality of life [6, 7], and all-cause mortality [5, 8, 9]. From an economic perspective, it is estimated £7.4 billion is spent per-annum on the consequences of inactivity within the UK alone [10]. For these reasons, addressing the complex behavioural challenges surrounding inactivity has remained a concern for regional and national stakeholders and policymakers for upwards of 60 years [11–14].

Historically, population-level interventions, programmes, and schemes aiming to improve PA participation have reduced implementation to parsimoniously modifying single or multiple factors on the individual (e.g., motivation), interpersonal (e.g., social support), or environmental (e.g., access to facilities) level in an effort to elicit change in the *‘intention’* of behaviour [15–17]. Although interventions defined to a specific *‘place’*, *‘setting’* or *‘population’* and underpinned by strong participatory research and behavioural theory have shown promise in modifying behaviour over the short- to medium-term, these efforts often require high agency, exacerbate inequalities, and report poor long-term acceptability, feasibility and effectiveness at a population-level [16, 18]. In addressing this challenge, more recently, public health policy approaches (e.g., Uniting the Movement, Local Delivery Pilots) have moved towards *‘whole systems-based’* thinking, methods, and place-based working practices to try to change and sustain population PA behaviours [19–21].

Systems-thinking represents a broad set of approaches which rather than limit the intricacy underpinning a behaviour, seek to understand, embrace, and challenge its complexity [19–21]. More specifically, these approaches consider PA as a product of a dynamic system (e.g., PA behaviour changes over time due to factors like seasons or unexpected events). The system is also adaptive (i.e., it is not constant, and new factors emerge, continually changing how they interlink), non-linear (e.g., the relationship between one factor such as a policy designed to create opportunities, does not consistently or equally affect another such as reducing inequality), and uncertain (e.g., PA behaviour cannot be directly predicted). This system is represented by a series of interdependent behavioural factors (e.g., individual, interpersonal, social actors, political, structural, and environmental factors) across multiple levels of influence [19–21]. Central to systems-thinking, above non-linear *‘cause and effect’* behavioural theories that focus on the *‘intention to change’*, is the concept that the whole-system is adaptive. This means that it is influenced by feedback (i.e., outputs of the system such as improved health or PA participation, create inputs within the system, which in turn influences how it functions), interventions and actions within the system, the social power of actors, and structural changes within and outside to its boundaries [20, 21].

Systems-maps are a commonly adopted tool to illustrate this nuanced complexity [20, 22, 23]. More specifically, these maps provide visual insight into how factors across the systems influence each other [20, 22, 23], assist in the identification of points of leverage [24–26], and complement evaluation and monitoring [27, 28]. Good evidence [23, 26, 29, 30] indicates maps can be produced from a variety of primary and secondary forms of qualitative (e.g., workshops, interviews, focus groups, co-production, reviews) and quantitative (e.g., surveys, analysis) sources of data. Notwithstanding, evidence suggests adopting methods which are participatory, may produce additional benefits such as building a shared agreement on the nature of the problem, identifying roles within the system, and co-producing policy and strategy responses [20, 29]. Albeit mapping cannot involve all people involved or affected by the system, good evidence [23, 31] indicates any process designed to map the system should include a diverse range of participants (e.g., stakeholders, deliverers, policymakers, people with lived experience) to provide a nuanced understanding of the system, its behaviour, and dynamics.

Within any system, leverage points are present [24, 25]. These are factors which are vital for meaningful change, and which if modified can impact upon the function of the system [24, 25]. To assist stakeholders, policymakers, and researchers to understand and identify different types of leverage points, various models, theories, and frameworks exist, which are not limited to the Meadows 12 [32], Public Health 12 [25] and Action Scales Model [24], and various markers from social network analysis [33] (e.g., degree, betweenness, eigenvector). Broadly, leverage points are identified, formulated, and evaluated to the extent in which they influence the system [24, 25]. Across multiple models, leverage points can be clustered into a hierarchy. Paradigms and beliefs refer to deeply held philosophies at the foundation of the system, such as the value of movement and the mental models on how the system ‘*looks*’ or ‘*is*’. Goals involve the system’s purpose, targets, and ambitions such as broad aims to increase movement. Systems structures encompass environmental, social, economic and infrastructure such as low traffic neighbourhoods, and how these factors interact and shape how the system functions. This may include how information flows within the system or feedback loops, which describe how an output of the system such as changes in health, influences its own input (current state of health) either by amplifying or balancing. Finally, events are the symptoms or outcomes of the system (e.g., behavioural interventions – daily mile, cycle to work schemes) [20, 24, 25, 32]. Across this hierarchy, leverage points which modify paradigms, attitudes, norms, and rules (i.e., beliefs, goals) or the dynamic structure of the system and its information flows and processes offer a culture change and therefore greater leverage [24]. In contrast, leverage points such as interventions (e.g., events) are often static within the system, operate in isolation and therefore only provide short-term solutions to problems present within the system, and not wider systems-change [24].

Global (i.e., WHO Action Plan, Eight Investments Which Work) [13, 34], UK (i.e., Uniting the Movement) [35], regional and local (e.g., Suffolk Core20PLUS5 [36] policy and evidence [19] emphasises the importance of place-based (e.g., at a county-wide level) systems change when attempting to improve population PA participation. Suffolk, UK is one example of a place-based system, whereby defined regional geographical and policy boundaries exist [36]. The county is a disparate mix of rural (i.e., village) settings, amongst coastal and high-density urban dwelling population hubs (e.g., Ipswich, Bury St Edmunds) [36]. Amongst differences in geography, there are meaningful health inequalities such as variation in age, multiple deprivation, life expectancy, access to health services, housing, and education across the county [36]. Moreover, Suffolk has disproportionately greater mortality related to cardiorespiratory events, poor mental health, COPD, and cancer [36], an increasingly aging population, and reduced healthcare provision, infrastructure, and investment [36].

This interlinking complexity of compositional (individual-level), contextual (environmental-level), and wider (systems-level) factors unique to Suffolk emphasise the importance of the prevention of conditions via lifestyle-based health behaviours tailored to the place (e.g., PA) [19–21]. Indeed, current regional government strategy stresses the need for direct and indirect support for population change in lifestyle behaviours [36]. This need and complexity underscore the importance of systems-thinking and whole-systems working across the county [19–21]. For this reason, our research team began working with Active Suffolk (www.activesuffolk.org), the Active Partnership (see www.activepartnerships.org), to develop a systems-map that represents PA participation in Suffolk and identify leverage points for change. This was a process which stakeholders or policymakers across the county were yet to explore.

## Objectives

The objective of this research was to explore the complexity surrounding insufficient PA across Suffolk through a systems-thinking approach by: (i) understanding what determines participation in PA in Suffolk (ii) mapping the system and (iii) identifying leverage points within it and (iv) inviting partners to develop solutions and actions points for change. To deliver these objectives, we worked with a consortium of partners across the PA and public health landscape in the county.

## Methods

### Design and overview

**Fig. 1:**
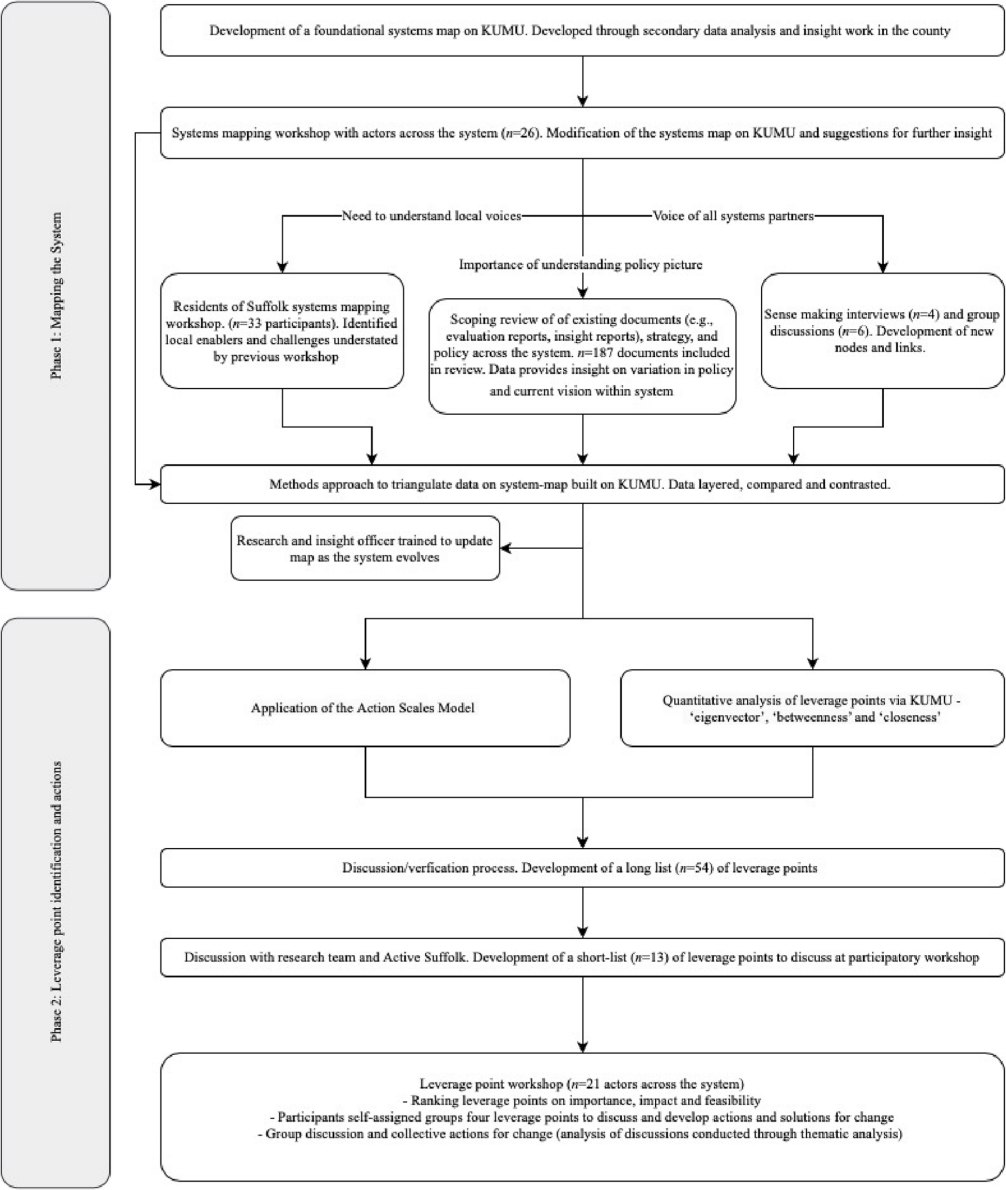
Visual overview of research design

To determine the factors influencing participation in PA, formulate the system and identify leverage points within it, we worked alongside Active Suffolk to develop a broad consortium of partners. These participants represented (i) regional government and public bodies, (ii) local government and public bodies, (iii) regional PA stakeholders and charities, (iv) deliverers and provisions, (v) advocacy groups, (vi) participant representatives, (vii) primary, secondary and public health bodies, and (viii) education and young people. The consortium engaged in two phases of the research to varying degrees (Fig. 1), and was communicated with throughout the project (e.g., regular updates on systems-mapping process). Phase 1 sought to understand the factors and map the system that underpins PA participation within the county. Phase 2 identified leverage points and actions via a mixed-methods analysis and a group workshop. Ethical approval was provided by the University of Essex (ETH2223-2022). This research conforms to, and was conducted in accordance with, the Declaration of Helsinki. All individuals involved in primary research (e.g., workshops, interviews, discussions) were considered study participants and provided written informed consent to participate in the research.

### Systems-map development (Phase 1)

#### Overview and systems-map development

To understand the complexity underpinning PA participation (i.e., walking and cycling for transport, sport, exercise, play and leisure) we utilised a pluralistic [37] sequential process, using a *‘methods approach’* to triangulate data into our [38, 39] conceptual systems-map (constructed via KUMU™). Conceptual systems maps illustrate the complex relationship between a range of factors and can assist stakeholders and policymakers to respond to problems, design solutions, and effect change [20]. Multiple-methods were used to identify and link factors within the map from varying perspectives [38, 39], complementing philosophies that systems are non-linear, adaptive, and a changeable phenomenon. An initial foundational map was developed and generated on KUMU™ by AB and KC, and critically appraised by the broader research team (RSE, PF, VG). This was informed by identifying and linking factors present within the existing literature [19, 20, 22, 40–43], a descriptive analysis of the 2021 Active Lives data [2], and insight work (e.g., small-scale evaluations and projects, such as GAPPA mapping [13, 34]) and ripple effects mapping [44] conducted within the system). This map formed a foundation for participatory mapping workshops, a narrative review of evaluation reports, policy and strategy, and interviews. Further, given systems are non-linear, adaptive, and changeable [19–21], we additionally trained one insight officer (Active Suffolk) in understanding the system and how to update the systems-map via KUMU™.

This approach enabled over multi-iterations to layer, compare, and contrast data, which provided insight into divergence, inconsistency, or deficiency in the map [19–21, 39, 45]. Completed by AB and KC in the first instance, and critically appraised by the broader research team at each stage, this provided understanding into where additional data would provide greater clarity on a given factor or link. Where such a complication arose, additional data was collected to remove this paucity. To build our map we utilised inductive reasoning to identify, specify, and critically appraise factors (e.g., circles on map) and the links between these.

#### Participatory systems-mapping workshops and sense making

To evolve the systems-map to a local context, we conducted two systems-mapping workshops. First, a half-day face-to-face participatory systems-mapping [23] workshop was conducted with a sub-set of individuals across our consortium of partners, to explore the initial factors influencing PA participation (workshop 1). Participants (*n* = 26) were sampled to represent the heterogeneity of organisations and roles involved in the consortium and invited via email. Participants represented regional government and public bodies (23%), local government and public bodies (7.5%), regional PA stakeholders and charities (4%), deliverers and provisions (12%), advocacy groups (15.5%), participant representatives (7.5%), primary, secondary and public health bodies (23%), and education and young people's organisations (7.5%). The participants were from a variety of organisational roles. Specifically, participants were CEOs (20%), directors/heads (15%), managers (57%), and officers (8%). During the first workshop, participants were introduced to systems-thinking, its application within PA contexts, basic terminology (e.g., factors, connections), and the foundational map (mentioned above) and its key features. Following this, in smaller groups (*n* = 4), participants were provided with a copy of the foundational paper (A0 size), and worked through a four-step sequential process to modify each factor, link, and theme (i.e., group of factors).

More specifically, participants first discussed the key enablers and challenges to PA participation within Suffolk, and amended their map as required. Following this, participants drew and amended links between ‘factors’ on their map and had the opportunity to review other maps within the workshop, and further refine their map. Examples of modifications included amending connections between stakeholders and factors reflecting infrastructure, while additions included a greater awareness of rural-specific challenges (e.g., the absence of an effective transport network). Following a final discussion of the maps and key features of these, participants suggested additional data and mapping were required to; (i) understand the enablers and challenges unique to Suffolk; (ii) perspective of residents across the county.

To deepen local understanding, and consistent with good evidence [23, 46] we conducted an independent systems-workshops with residents of Suffolk. We adopted a modified version of our initial systems-workshop to deliver a half-day face-to-face participatory systems-mapping [23] workshop to understand the factors of participation from the perspective of individuals residing within the county (workshop 2). The workshop was coordinated by a local charity and represents a practical approach to involving community members in real-world research. More specifically, we used convenience sampling to recruit, working-age and older-adults (*n* = 33; Age: 65.7 ± 9.8, 45% females) who represented a range of social-demographic factors (e.g., deprivation, living arrangements) and localities to a systems mapping workshop. Unlike the workshop one, we adopted a simplified sequential process to development. Working in small groups (*n* = 5–6) and on a sheet of A0 paper, participants defined PA. This definition formed the centre of map. Following the process, participants listed PA enablers and challenges around their definition. Organically, and following direction from a facilitator, participants linked these enablers and challenges (e.g., crime linked to feelings of low self-confidence exercising in green space). This exercise of linkage created further conversations, and the development and adaption of additional factors. Finally, participants were invited to visit other maps and discuss the challenges and enablers they faced. This process led to further revision of the maps. Maps from workshops 1 and 2 were collated and layered into the broader systems-map for Suffolk by AB and KC following workshop two.

Systems-workshops are limited by the extent of the individuals and organisations present [23]. For this reason, the research team conducted interviews (*n* = 4) and small-group discussions (*n* = 6) with partners across the consortium who were willing to participate in, but unable to attend workshop 1, to make sense of the system, identify additional factors of participation, and gain feedback on the systems-map. These sessions lasted about one-hour, were conducted via online video communication software (Zoom™) or face-to-face and involved between three and eight partners in the case of group discussions. Within each session, the researcher provided an overview of the map, an insight into the functionality of KUMU™, and themes within the map (e.g., built environmental factors). Following this overview, participants were asked to reflect on how the map represented; (i) their organisation and its function/operations; (ii) Suffolk broadly. Critical feedback provided insight into the creation of new factors, and revision of existing factors within the map. Data were transcribed verbatim, deductively coded, and layered onto our evolving systems-map by AB and KC [38, 39].

#### Document, policy, and strategy review

To expand on our understanding of the system we (AB and KC) conducted a narrative review of existing documents (e.g., evaluation reports, insight reports), strategy, and policy across each of the systems sectors outlined above. To identify grey evidence, we used Google Scholar, Overton, and independent searches of each systems-partners' website. Due to relevancy, searches were limited to 2010 to date, and included search terms formulated from the foundational systems-map, in combination with regional locations and PA (see additional file 1). To expand our review, indexing (i.e., searches for documents within included evidence) and sibling-searches (i.e., related evidence conducted by the same author/organisation) were conducted. For inclusion, evidence had to be (i) presented within written, audio, or visual format; (ii) be related to PA; (iii) be related to Suffolk. In total, 187 documents were included within the review. Data representing factors within the system (e.g., existing policies, strategies, evidence of systems-partnerships) were extracted from the included documents. This evidence was deductively coded against the existing system-map [38, 39].

### Leverage point identification and actions (Phase 2)

#### Initial analysis and framing workshop three

There remains no gold-standard to identify and specify leverage points within a system aiming to understand a public health phenomenon [47]. Therefore, to identify leverage points with our system, we utilised a sequential quantitative, qualitative and participatory approach, which served as a precursor to a workshop with consortium members (workshop 3). Similar to previous approaches [26], we first adopted quantifiable leverage measures [33] to unpick the complexity of our map. Network analysis of systems-maps can result in false inference [48], therefore, we were cautious not to use these metrics to confirm leverage points, but rather to assist in the broad identification of places to consider modifications to the system. With this in mind, we used the KUMU™ in-built metrics tool on the ‘factor level’ to analyse factors based on their *‘eigenvector’* (i.e., how well connected a factor is with other well-connected elements), *‘degree’* (i.e., the number of connections), and *‘betweenness’* (i.e., its role as a bottleneck between factors). This helped us evaluate the likelihood that a factor might be useful for leveraging change or could pose challenges to this [33]. Following the identification of up-stream factors (i.e., factors which could result in broader systems change, such as policy, relationships, sharing resources/knowledge) with a high eigenvector, betweenness, and degree values, we applied the Action Scales Model [24]. This was selected due to its parsimony, accessibility, and our need to translate findings to systems-partners in a clear and understandable style [24]. This conceptual tool integrates the complexity of various models, tools, and frameworks, such as the Meadows 12 [32] (and the translated public health version – ‘Public Health 12’ [25]), alongside the Iceberg Model [49] and Intervention Level Framework [50]. The model indicates leverage can be conceptualised into four progressively influential points of change (see Nobles et al. [24] for a more detailed overview), specified as; *‘events’* (i.e., the outcomes, behaviours, symptoms of the system); *‘structures’* (i.e., the environmental and social factors which shape events within the system, and the relationships, patterns information flow within the system); *‘goals’* (i.e., the rules, policies, strategies, and ambitions within the system); ‘*beliefs*’ (i.e., deeply held values, norms, and attitudes within the system).

The analysis process was sequential and conducted independently by three members of the research team (AB, KC, RSE). Researchers met to critically discuss each of the leverage points within the system. These discussions were expanded with the broader research team, whereby an internal verification process was carried out and a long-list of 54 leverage points was developed. Following these steps, this long-list was discussed and reduced to 27 leverage points by three members of the research team (AB, RSE, PF). Following this process, two members of Active Suffolk staff immersed in the system with local insight and understanding assisted the research team in further specifying points to leverage change within the system. Leverage points within the long list were debated on the extent in which they held the capacity to cause larger systems change. For the purposes of the leverage point workshop (workshop 3), we outlined 13 points within the system where change may be effectively leveraged.

#### Leverage point workshop (Workshop 3)

Workshop three built upon the initial analysis and invited participants to rank leverage points and develop strategies, methods, and approaches to leverage change. Participants involved in the workshop were sampled to represent the range of organisations across the Suffolk PA system and were invited to a half-day leverage points workshop via email (n = 21). 30% of participants who attended the first workshop also attended the second workshop. These participants were sampled from regional government and public bodies (15%), local government and public bodies (35%), regional PA stakeholders and charities (15%), deliverers and provisions (20%), and primary, secondary and public health bodies (20%). Participants represented a variety of roles. More specifically, CEO’s (5%), directors/heads (14%), managers (60%), and officers/deliverers (21%) were involved in the identification and development of leverage points.

Delivered in sequential steps, the research team provided an overview of the system representing PA within Suffolk, key features of the systems-maps (e.g., themes), leverage points, the Action Scale Model [24], and the analysis of leverage points conducted prior to the workshop. Following this overview, in three groups of seven, using cards, participants were asked to rank leverage points considering their; (i) feasibility; (ii) potential impact; (iii) importance within Suffolk. The research team collated this output and outlined the top four leverage points to modify within the county (i.e., based on total sum of importance). Following this, participants self-assigned themselves a leverage point to discuss, develop solutions for, and modify (four groups of *n* = 4–6). Supported by a facilitator from the research team and inspired by the process outlined within the Action Scale Model [24], participants considered how, where, when, what, and why changes should occur. Finally, participants presented their solutions to the broader group, and the context and considerations this operates within. This was followed by questions and debate by the broader group. These presentations and conversations formed the basis of action points for on-going systems change (noted on post-it-notes). Conversations and observations by the research team were recorded via voice recorder or notes. These were transcribed verbatim, and analysed through a deductive thematic analysis [51]. Finally, following the workshop, we analysed the correlation between combined feasibility and impact scores, impact and importance, and feasibility and importance scores using Spearman rank correlations.

## Results

### Phase 1: Overview of the systems-map

**Fig. 2:**
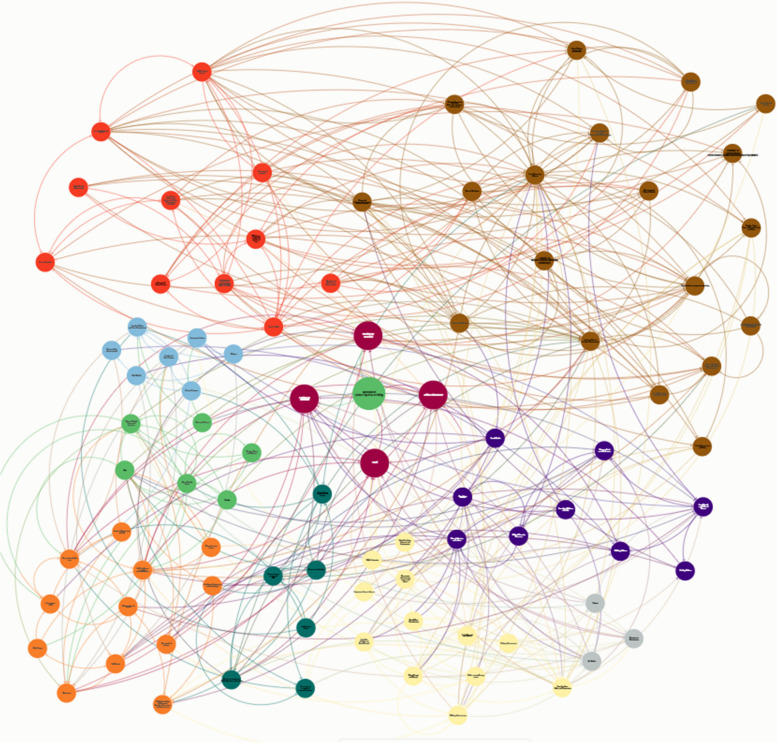
Systems-Map Representing Physical Activity within Suffolk. *Notes:* Biological individual factors (green), financial individual factors (dark teal), psychological individual factors (light orange), interpersonal factors (light blue), systems partners (dark orange), built environmental factors (light yellow), natural environmental factors (light grey), social environmental factors (purple), and policy and structural factors (brown), individual modes of physical activity (red), total activity (green)

An overview of the systems-map is provided in Fig. 2, with an interactive version accessible on KUMU™ (www.tinyurl.com/SuffolkPA) including definitions for each factor and the causal links between these. To understand the causal links between each factor in detail, we encourage readers use our KUMU™ version. The map is constructed from 90 factors that are segregated into nine themes (biological, financial, and psychological individual factors, interpersonal factors, systems partners, built, natural and social environmental factors, and policy and structural factors). These factors influence four individual modes of PA (cycling, walking, active leisure/play, sport), which contribute to overall PA.

Within the system, feedback loops are present. Whilst there is not scope to discuss each of these in sufficient detail within the present paper (we encourage readers to access KUMU™), a notable example does include active transport. Indeed, in the case of active transport, a positive (reinforcing) feedback loop exists, whereby the observation of walking and cycling within a population shapes the attitudes of stakeholders and residents that active transport is a safe and engaging activity (i.e., a walking culture). A positive culture towards active transport influences the formation of policies and strategies (i.e., goals in the system), which shape broad urban planning regulations (i.e., a structure). These regulations determine cycling or walking infrastructure (i.e., structure), which finally increases observed participation in walking or cycling (i.e., an event). This ‘loop’ in many senses is consistent in terms of each mode of PA, however, expressed differently due to time delays (e.g., seasonality, allocation of funding, time for policy to have effect), and differing policy, and built, social and natural environmental factors within the system (e.g., rurality, resources, relationships).

### Phase 2: Overview of leverage points and initial analysis

A complete presentation of each factor, and its respective eigenvector, degree, and betweenness is available via KUMU™, within the ‘table’ function. Grouped across levels of the Action Scales Model [24], Table 1 outlines the twenty most influential factors ranked on eigenvector (excluding modes of activity). In addition, critical discussions based on the Action Scales Model [24] additionally highlighted ‘urban design and density’, ‘speed volume of traffic, driver behaviour and culture’, ‘multiple deprivation and socioeconomic status’, ‘cycling culture’, ‘organisational values and beliefs’, ‘funding (e.g., health, fitness professionals’ schemes, interventions, programmes), and ‘social media’ as important factors for leverage within the system. Table 1 outlines why each factor was selected. Critical discussions between the research team and Active Suffolk against each leverage points feasibility, impact, and importance within Suffolk led to the following factors being brought forward to the leverage point workshop designed to combine scientific research with local systems-knowledge and insight.

**Table 1:**
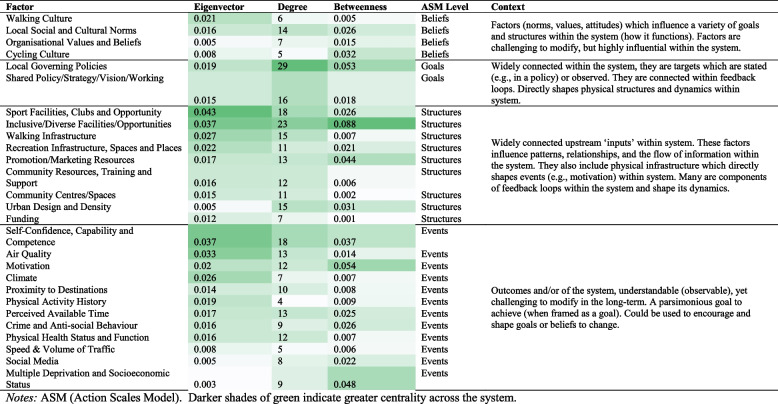
Leverage point analysis and selection

### Phase 2: Findings of leverage point workshop (Workshop 3)

#### Ranking leverage points within the system

Following critical discussion within groups, participants ranked leverage points within the system on their feasibility, impact, and importance (Table 2; see Attachment 2 for a more detailed overview). Group rankings across each leverage point and the total sum of importance were used to underpin the top-4 solutions below. Leverage points of seemingly greater impact (Median: 19, IQR: 12) were ranked poorly in terms of feasibility to change (Median: 20, IQR: 12), but not significantly (rho = -0.20, p = 0.49). Further, leverage points with high importance (Median: 17, IQR: 12) typically represented a greater degree of leverage within the Action Scales Model [24], but were not correlated with feasibility (rho = 0.42, p = 0.14) or impact (rho = 0.39, p = 0.17). Observations from the research team indicate participants representing strategic organisations or involved in policymaking tended to preference ‘beliefs’ and ‘goals’, whilst organisations with a meaningful delivery capacity placed emphasis on ‘events’ within the system.

**Table 2:** Leverage points from workshop ranked on impact, feasibility and overall importance

Leverage Point (Ranked by Total Importance)	ASM Level	Total Scores		
		***Importance***	***Impact***	***Feasibility***
1. Local Governing Policies	Goals	11	14	20
2. Shared Policies, Strategies, Vision, and Working Relationships	Goals	13	17	12
3. Shared Facilities (School, Sport, Community, Recreation)	Structures	14	21	19
4. Funding	Structures	15	19	25
5. Inclusive/Diverse Facilities/Opportunities	Structures	16	32	13
6. Self-Confidence, Capability and Competence	Events	16	11	26
7. Recreation Infrastructure, Spaces and Places	Structures	17	11	20
8. Local Social and Cultural Norms	Beliefs	21	15	33
9. Community Resources, Training and Support	Structures	22	22	14
10. Promotion/Marketing Resources	Events	27	36	3
11. Walking Culture	Beliefs	30	15	24
12. Cycling Culture	Beliefs	33	21	29
13. Crime And Anti-Social Behaviour	Goals	38	39	35

*ASM* Action Scales Model. Total scores are a sum of the rankings from each group. Leverage points are ranked on importance. Lower scores indicate greater importance, impact, or feasibility

#### Developing solutions to leverage change, actions, and next steps

**Fig. 3:**
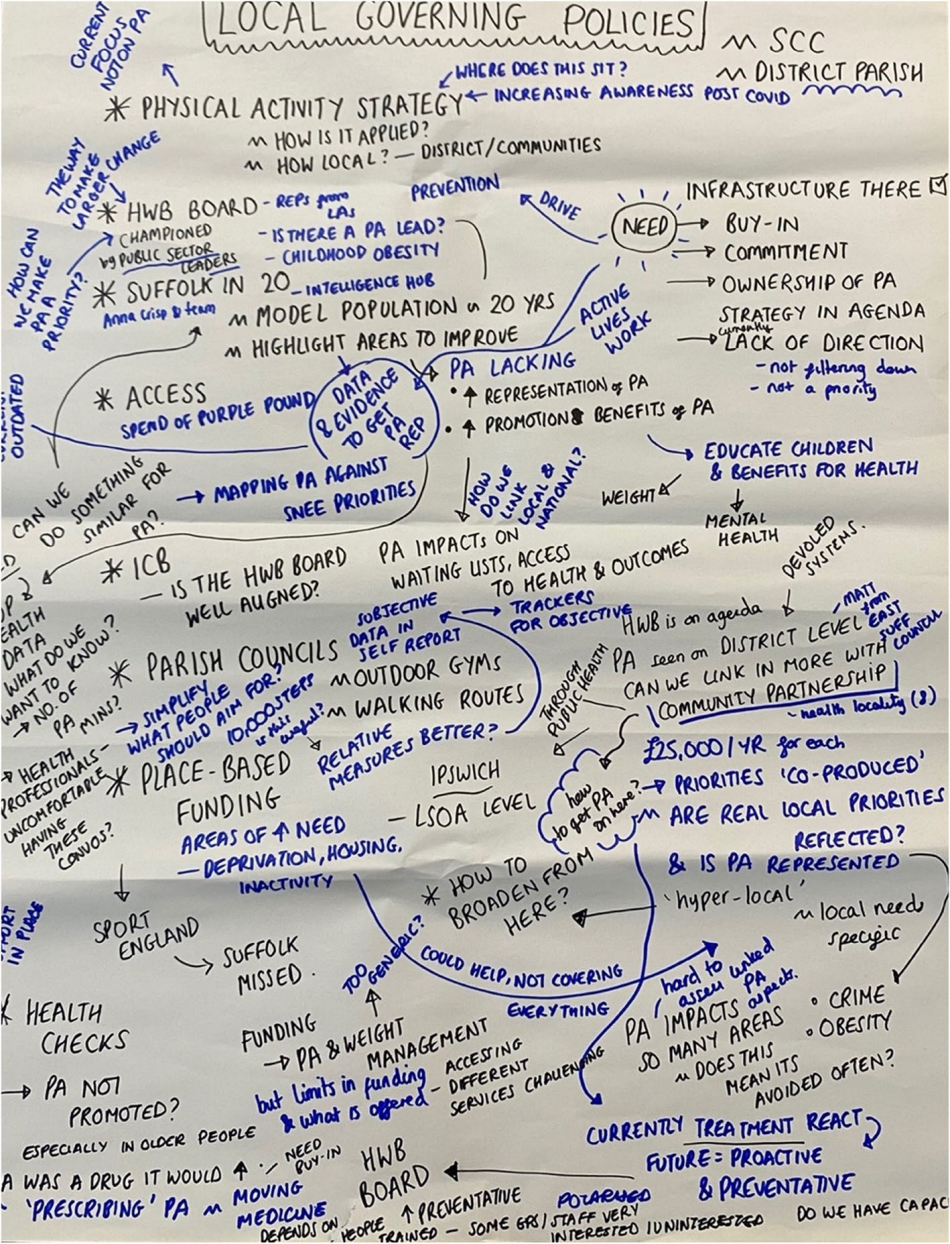
Discussion Workshop Poster. *Notes:* Discussion poster from group focusing on local governing policies

Solutions related to (i) local governing policies, (ii) shared policies, strategies, vision, and working relationships, (iii) shared facilities (school, sport, community, recreation), and (iv) funding were developed by four sub-groups. An example of a group’s solutions, challenges, context, and considerations when leverage change is outlined within Fig. 3.

#### The importance of collective working, relationships, vision, and strategy

Within the broader group discussion in workshop 3, participants acknowledged the importance of shared working relationships, a collective vision, and strategy. Whilst not complex to establish such relationships or a core ‘vision’ (i.e., to get people active) within the consortium, it was deemed challenging to get organisations to functionally work together in the long-term (e.g., creating a shared strategy on PA).

Central to this challenge was that organisations often held divergent models of delivery, organisation, or policymaking that frequently conflicted or challenged each other. In proposing a solution to this challenge, one participant discussed the importance of *‘collective buy-in’* where all deliverers, organisers, unexpected actors, and public health partners are provided a space and voice to co-produce strategic goals and collaborate on a shared strategy:

‘It's about collaboration. It's about understanding how we can all contribute. It's about understanding that shared vision, shared goals and recognizing that everybody has something different to add’. *Senior Policymaker (Stakeholder)*

#### A co-produced strategy and vision

Any co-produced strategy should be applicable at a local, district/ward and regional level, and a product of shared evidence. One solution presented by a group was a 20-year prediction of health and wellbeing in the county. In such evidence, a scenario would be presented whereby the current predicted state of health would be compared against a 20-year plan with collective change and direction surrounding PA (i.e., mapping PA within the priorities of the integrated care system). The use of shared evidence and proposed collective working was deemed essential in the ownership and function of a whole-systems approach. This *‘golden thread’* of evidence was deemed important given: (i) it raises the priority of PA within public health conversations; (ii) current health and wellbeing policymakers such as the integrated care system/board operate in a 20-year timeline currently. Central to engaging such policymakers such as regional health and wellbeing boards with any vision or strategy was switching the delivery and organisation model from being reactionary to preventative (i.e., not reacting to events in the system, but adapting structures, goals, or beliefs):

‘It’s about switching the emphasis to prevention. I'm preaching to the converted, but prevention rather than reacting to the problems that we've got. We felt perhaps in the physical activity strategy there could be a shared vision that is underpinned by some principles and evidence that everyone could buy into. That would be good’*. Senior Operations Director (Local Council)*

#### Sharing facilities and resources

A challenge related to evidence, knowledge, and insight was highlighted by the group focusing on shared facilities. Due to its rural and expansive nature, Suffolk is a region with a large quantity of facilities, spaces, and places to participate in PA. Often owned or managed by schools, education providers, councils, and unexpected providers (e.g., religious groups), there remains no collective audit or database of such facilities. An area, the group acknowledged was important in terms of a collective strategy surrounding the shared use and management of facilities. With such a strategy, participants discussed the importance of supporting unexpected providers with funding and resources to open outside of normal-working hours:

‘There are facilities that are there that are probably underused. So we're talking about school halls, not necessarily sports centres as such, but school halls, church halls, community facilities, things like that, that need to be used, but they probably have a little bit of an issues. They don't know how to open up, or they don't have the resources to open up. We talked about what do we want to get from those facilities? So is it traditional sport? Is it physical activity? Is it really, really soft approaches? We then focus a lot of our time onto the strategy stuff. So can we produce? Suffolk wide strategies that help for those facilities to open. So is there something to not necessarily force facilities into opening?............But actually, if we can force that happen with a collective approach, but this is actually going to be mutually beneficial. *Regional Sports Provider (Regional Governing Body)*

This underscores the importance of inviting the owners, managers, or administrators of such facilities to any level of strategic development and consortium.

#### Funding

Funding was deemed as a key leverage point within the system. For example, participants acknowledged for any meaningful change to occur in PA behaviour, the way in which funding is allocated and spent across systems partners needed to be modified. Indeed, funding for PA, was deemed to focus on short-term projects (e.g., events in the system), be reactionary, rather than preventative, and rarely collaborative. To many extents, partners commented that this approach to funding within the system, promoted competition among organisations, rather than encouraging whole-systems working. Consistent with other leverage points, a collective vision and collaborative model was proposed. This centred on long-term pooling resources, shared capacity, and collective investments. Key was funding *‘what works’* and acknowledging *‘what doesn’t*’ through shared insight, evaluation, and monitoring:

‘Sharing what we do and then agreeing to replicate our work with each other, to in a sense, expand the projects we're working on, across and between the district boundaries. If we decide to work like that, we could do it. It could be really exciting’.* Operations Manager (Stakeholder)*

#### Solutions and actions points to promote change

Actions signalled steps for individual, organisational, and shared commitments to leverage change (see Tables 2 and 3). Broadly, these ‘actions’ reflected a need to leverage change within ‘goals’ in the system, such as sharing policies, resources, insight, evidence, and capacity, working together on projects and funding applications, and collaborating to co-produce a collective vision and strategy. Actions also reflected a need to raise awareness, build an evidence base, create a step change in the priorities of public health (i.e., moving PA up the agenda) for strategic policymakers (e.g., heads of local government, integrated care system and/or board).

**Table 3:** Actions for change

Action (Type of Organisation)	Type of Organisation	Leverage Point	ASM Level
Include physical activity, exercise and/or sport within strategy or priorities	Deliverer/organiser	Local governing policies	Goals
Collaborate with other districts, organisations, and wider partners– share information about projects and grant funding	Organiser	Shared policies, strategies, vision, and working relationships	Goals
Shared vision, direction, relationships, and ethos within and across organisations	Stakeholder	Shared policies, strategies, vision, and working relationships	Goals
Understand, advocate, and support the use of shared spaces, places, and facilities	Stakeholder/deliverer	Shared facilities	Structures
Promote ‘work’ and good stories across county	Stakeholder	Shared policies, strategies, vision, and working relationships	Goals
Champion insight and impact	Stakeholder	Shared policies, strategies, vision, and working relationships	Goals
Advocate for physical activity across the county	Stakeholder	Shared policies, strategies, vision, and working relationships	Goals
Develop an advocacy/support group for policymakers	Stakeholder	Shared policies, strategies, vision, and working relationships	Goals
Develop a volunteer and resident voice group for physical activity across the county	Organiser	Shared policies, strategies, vision, and working relationships	Structures
Share knowledge and skills with other organisations to improve access	Organiser	Shared policies, strategies, vision, and working relationships	Structures
Engage, challenge and work with the integrated care system (NHS) to build advocacy for physical activity	Stakeholder	Shared policies, strategies, vision, and working relationships	Goals
Provide collaborative leadership on funding	Stakeholder	Funding	Structures
Coordinate a countywide physical activity strategy	Stakeholder	Shared policies, strategies, vision, and working relationships	Goals
Develop ‘future’ evidence/position – Suffolk in 20 years report	Organiser/stakeholder	Local governing policies	Goals

*ASM* Action Scales Model. Actions are grouped into common themes. NHS (National Health Service)

There was a theme of influencing *‘good will’* or *‘hearts and minds’* of these policymakers via promoting the success of collaborated projects (e.g., fit villages), and a shared voice, vision, and strategy for the future. In addressing this, the consortium underpinning the current project was proposed as a solution to create advocacy. However, although this group has the positives of a flat hierarchical structure (i.e., equal partners), it was acknowledged that there remains no leader managing and coordinating the system.

## Discussion

The objective of this research was to explore the complexity surrounding insufficient PA participation across Suffolk through a systems-thinking approach. We did this through two phases of research, whereby we mapped the system (Phase 1) to (i) understand what determines participation in PA in Suffolk and (ii) illustrate its complexity. Within Phase 2, using a participatory approach, with a consortium of stakeholders, deliverers and policymakers, we used our systems-map to (iii) identify leverage points and (iv) used these leverage points as starting points to develop solutions and actions points for change.

Within Phase 1, a conceptual systems-map of the factors shaping PA in Suffolk was developed. This comprised of 90 factors that converged around nine themes. Through a participatory approach within Phase 2, we identified the four most important leverage points for change were: (i) local governing policies; (ii) shared policies, strategies, vision, and working relationships; (iii) shared facilities (school, sport, community, recreation); and (iv) funding. Factors which were linked within feedback loops within the system. Actions to leverage change within Phase 2 included raising awareness with partners beyond the system, sharing policies, resources, insight, evidence, and capacity, and collaborating to co-produce a collective vision and strategy. These findings complement and extend the growing body of evidence, which has utilised a pluralism of methodologies to understand the dynamic system underpinning participation in PA [19–21] and chart leverage points for change within this complexity [22, 40–42, 52, 53]. This research provides important insight into the steps underpinning a shift towards a whole-systems and place-based approach to working. A point recently emphasised within an influential expert commentary [54].

Our map constructed within Phase 1 shares many of the same intrapersonal- (e.g., motivation), interpersonal- (e.g., social support), environmental- (e.g., facilities) and policy-factors (e.g., funding) identified at a global, national, and regional-level within systems- and ecological approaches aiming to understand PA behaviour [20, 22, 40–43, 52, 53]. Moreover, many of the same organisations present within the policy space surrounding PA (e.g., regional government, healthcare organisations, providers) [52] were present in our analysis, as were the connections between these organisations [42], and the themes in which policy is shaped around [40] (e.g., environment, society, systems, people). However, a system is a dynamic, adaptive, time-variant and emergent web [19–21], which responds to feedback, and is shaped by factors internal and external (e.g., national governmental policy) to its boundaries [20]. Therefore, although systems-maps within a context (e.g., PA promotion) share many similarities [20, 22, 40–43, 52, 53], how factors interact [19–21], how change is intentionally and unintentionally brought about, and how the social power of actors influences change is unique [20, 21]. This places an importance on the application of systems-thinking approaches when addressing inactivity [54]. Moreover, this is particularly prominent in the case of leverage points [20, 24, 25, 32] (Phase 2), where when using a participatory approach we found the extent in which change can be influenced is underscored by its feasibility, perceived importance, and potential impact by actors within the system.

Interestingly, in all cases within Phase 2, participants ranked leverage points which reflect goals or structures (e.g., policy, resources) in the Action Scales Model [24] as the most important to change. Modifying goals or structures within the system, is thought to influence larger whole-systems change (e.g., changing a policy has an impact on many, rather than a few) as they shape how the system functions and its dynamics [24, 25]. However, leverage points related to beliefs within the system such as cultural change, paradigms, or norms, were more often ranked poorly in terms of their feasibility to change. In agreement with Power and colleagues [42], changes upstream within the system such as a shift in paradigms, norms, culture and the mental models we hold in how the system is constructed may take sustained efforts, time and engagement [24, 25]. For these reasons, leverage points reflecting beliefs may appear to be unfeasible and low in impact to actors within the system. Therefore, it remains important for future research to unpick the long-term complexities of leveraging change within the PA space [55]. This is particularly important, given a shared belief in the system may support the likelihood of policies to be collectively shaped and implemented [24, 25] in the long-term.

Previous research seeking to encourage movement through a systems-approach found similar leverage points [52, 56]. For example, systems representing recreational participation have likewise found the importance of goals (e.g., serving the needs of young people) and varying forms of structural change (e.g., urban design, safety, policy change to support movement, laws, regulation) [52, 56–58]. These studies also underscore the importance of feedback loops and delays (e.g., appeal of facilities growing as more people use them) [52, 56–58], points in which the systems-consortium also recognised and discussed in regard to implementing a shared strategy. More specifically, consistent with research, it was recognised that it would take time to build consensus and an develop an effective whole-systems approach [17, 25, 59, 60], but a shared vision would bring in new partners, grow the system, and therefore the extent to which change could be brought about.

The current research identified several specific and important leverage points across the system which could enhance PA in Suffolk. Modifications to local governing policies, a shared strategy and vision, and collaborative working which transcend across the region, and into localities and districts of the county may have the capacity to cause downstream change to models of funding and the sharing of resources and facilities, points of leverage also identified within our research. However, the emphasis placed on these leverage points is consistent with the growing body of national and global strategies [19–21] and research [17, 25, 59, 60] which underscores the importance of whole-systems approaches in addressing complex societal complications and the importance of organisations working functionally in the long-term. The identification of these leverage points is also consistent with a recent evaluation of a whole-systems approach to promoting PA within an English county [41]. In addressing this challenge, research broadly indicates the importance of a central organisation to bring system actors together, drive change, and manage organisational differences, understanding the needs of each actor, their motivation and similarities in approach and strategy, and highlighting the co-benefits of systems-working (e.g., shared priorities) [41, 59, 61, 62].

In the case of our approach and sharing similarities with previous projects implemented within UK policy (e.g., local delivery pilots), this responsibility fell to the Active Partnership (i.e., Active Suffolk) [41, 63]. In agreement with Nobles and colleagues evaluation of *‘We Move Together’* [41], in the case of the present system, bringing participants together was acceptable and pragmatic, given there was already a broad emphasis placed on the importance of the needs for systems-based approaches surrounding public health and PA promotion within regional governmental policy (e.g., Core20PLUS5) [36]. Notwithstanding of this, participants within our research highlighted the absence of voices from within related areas of public health, and the broader integrated care system. The representation of voices, including the right people within a whole-systems approach, and exploring how these individuals interact (e.g., the strength of their relationships) has been previously acknowledged as a challenge to effective working and implementation [41]. Future research therefore may consider the application of modes of social-network analysis [23, 31] in exploring the interaction between actors across the system, and the identification of organisations not present within whole-systems approaches to promotion.

This research is likewise consistent with research unpicking whole-systems approaches, in that our participants emphasised rather than seeing divergence in strategy and policy, focus should be directed towards similarities and co-benefits [17, 25, 59, 60]. With our research, the concept of a shared vision, collective evidence base and strategy may also create a ripple-effect in that it reduces the clash of mindsets and ways of working observed in previous research [17, 25, 59, 60], and draws actors towards a central way of working, collective buy in, and ownership. This is particularly important in terms of funding and sharing resources and facilities across the county, where approaches were reported to be transactional, reactionary, non-collaborative, short-term, and focused on events within the system, rather than resourcing broader upstream changes [24–26]. These factors can promote competition and create divergence, rather than encouraging collective working [17, 40, 41, 61]. Consistent with previous research [17, 40, 41, 61], our research underscored the importance of funding and resource sharing through effective systems-leadership and collaboration on research and insight, advocacy, and knowledge and information sharing (e.g., resource availability, funding opportunities).

### Reflections and recommendations

This novel research adds to the growing body of evidence into using whole-system approaches to identify and leverage change to promote increases in PA. The following recommendations and actions are made based on the findings of this research:

#### Recommendations & actions

(i) A whole-system, participatory approach is required to ensure the correct people and local knowledge and insight are brought together to drive and advocate for change.
(ii) A shared vision is required to bring in new partners, grow the system and work towards long-term whole-system change. This will require time to identify and utilise the similarities between organisations and promote the co-benefits of working together.
(iii) Resources, such as system-maps that help to identify leverage points require regularly updating to reflect the dynamic and evolving nature of the system. This can be achieved through further engaging our systems consortium and a range of residents and populations across Suffolk.
(iv) Systems-mapping is an ongoing process. Our systems-map is limited in its ability to visually illustrate reinforcing and balancing feedback loops, and the dynamics within the system. Within the project, a causal-loop diagram will also be constructed using a participatory process. This will allow scope for systems-dynamics modelling, whereby factors and interactions across the model are explicitly modelled. Both options, may aid decision making within the system and reinforce the wider whole-systems approach. Indeed, through incorporating feedback loops, illustrating the evolution and unexpected dynamics of factors and their interactions within the system, and exploring simulated changes to leverage points (e.g., policies) stronger whole-systems change can be broad about.
(v) Individuals and organisations should be bold and open to not always doing the same and should not only consider leveraging change where it is most feasible but also where it may lead to the most impact.
(vi) It is important that policy to increase PA behaviour incorporates, encourages, and enables a system thinking approach.
(vii) In order for a systems approach to be embedded and sustained, it is important to train and upskill partners across the system in relevant approaches and tools (e.g., systems-mapping, identifying and modifying leverage points).

### Limitations and steps for future research

Though our research adds to the limited base of academic research seeking to explore whole-systems approaches within the promotion of PA, several methodological, pragmatic, and contextual reflections and considerations should be noted. Foremost, although network analysis can be a useful tool to identify leverage points, this should not be used in isolation [48], but in support of robust participatory approaches. Further, within our research and involvement in the broader system workshops, we adopted the Action Scale Model [24] over the Public Health 12 [25]. Whilst we found the Public Health 12 [25] to be an effective and nuanced tool in the identification and appraisal of leverage points, particularly in the case of detailing feedback loops, the parsimony of the Action Scales Model [24] proved to be useful as well. This model supported participants across the system, often without a grounding in behavioural science, in identifying and understanding leverage points throughout the system. We therefore recommend researchers consider the Action Scale Model [24] in their practice, whilst also utilising the Public Health 12 [25] as and when participants are confident in systems-based approaches and the identification of leverage points.

To some extent, this may be achieved by following our approach of training key participant(s) (e.g., research and insight officers) within the system. Whilst the acceptability and feasibility of this process is on-going, and not reported in this paper in detail, we have found this process to be useful in sequentially developing our understanding of the system and improving the knowledge of key organisations within it (e.g., Active Partnership, Local Government). Further, whilst a range of data sources contributed to the development (and maintenance) of our systems-map, consistent with a body of research [22, 23, 40, 42] we found participatory mapping to be the most useful and rich tool in both developing our understanding and our participants’ understanding of the system and its the nuanced complexity. However, in agreement with Cavill and colleagues [22] and Murphy and colleagues [40], we found this to be particularly effective when the process began with a foundational map and in our case was supported by multiple-methods [38, 39]. More specifically, sequentially evolving our map over-time as a response to weaknesses in the data proved to be useful in painting a comprehensive picture of the system which represents PA participation in Suffolk.

Finally, the next steps within our research and the system-consortium is maintaining momentum. Beyond the scope of reporting within the current paper, the systems-consortium, have begun to work on action points outlined within our leverage point, these are not limited to developing a shared vision and strategy and championing understanding of the system. One example of this is the recent Suffolk 2024–2027 *‘Move More to Feel Better’* PA and Movement Strategy [64], a policy, this research directly contributed towards. As highlighted above, a next step for the research is utilising social network analysis to understand and engage who is missing from the systems-consortium.

### Conclusions

This research adds to the growing body of evidence applying whole-systems approaches to the promotion of PA( 19–21). Our research demonstrates an approach to establishing a systems-consortium, developing an understanding of the system, and identifying leverage points within it. With context specific adaptions, our approach could be adopted by other actors seeking to adopt a whole-systems approach to change. Future developments with our system include growing and maintaining the systems-consortium, creating a sustainable means to continue mapping the system and identifying leverage points within it, implementing changes within the system, and monitoring and evaluating these modifications.

## Supplementary Information

Supplementary Material 1

Supplementary Material 2

## Data Availability

Our systems-map is publicly available on KUMU™ (www.tinyurl.com/SuffolkPA). The data used to generate this, previous iterations of the map, and data generated through the identification of leverage points, including our workshop is available via a request to the authors of the paper.

## Abbreviation

PA: Physical Activity
