# Supplementary material for: ‘It's about collaboration’: a whole-systems approach to understanding and promoting movement in Suffolk
Source: Int J Behav Nutr Phys Act. 2025 Jan 16;22:7. doi: 10.1186/s12966-024-01688-2 (PMC11740498; doi:10.1186/s12966-024-01688-2)
Supplement: Supplementary file 1 — Supplementary Material 1 [file 12966_2024_1688_MOESM1_ESM.docx]

**Additional File 1**

**Search Terms Used within Scoping Review**

Physical activity OR cycling OR walking OR sport OR play OR recreation OR active OR movement AND gender OR age OR health AND self-efficacy OR knowledge OR motivation OR habit OR value OR support OR family OR social OR parental OR history OR time OR commitment AND policy OR strategy OR plan AND transport OR car AND access OR culture OR crime AND behaviour OR facilities OR infrastructure OR design OR climate OR air
