# Supplementary material for: ‘It's about collaboration’: a whole-systems approach to understanding and promoting movement in Suffolk
Source: Int J Behav Nutr Phys Act. 2025 Jan 16;22:7. doi: 10.1186/s12966-024-01688-2 (PMC11740498; doi:10.1186/s12966-024-01688-2)
Supplement: Supplementary file 2 — Supplementary Material 2 [file 12966_2024_1688_MOESM2_ESM.docx]

**Attachment 2. Leverage Points from Workshop Ranked on Importance, Impact and Feasibility**

| **Leverage Point (Ranked by Total Importance)** | **ASM Level** | **Importance** | | | | **Impact** | | | | **Feasibility** | | | |
| --- | --- | --- | --- | --- | --- | --- | --- | --- | --- | --- | --- | --- | --- |
|  |  | ***G1*** | ***G2*** | ***G3*** | ***Total*** | ***G1*** | ***G2*** | ***G3*** | ***Total*** | ***G1*** | ***G2*** | ***G3*** | ***Total*** |
| 1. Local Governing Policies | Goals | 3 | 2 | 6 | 11 | 4 | 7 | 3 | 14 | 8 | 6 | 6 | 20 |
| 1. Shared Policies, Strategies, Vision, and Working Relationships | Goals | 9 | 3 | 1 | 13 | 2 | 11 | 4 | 17 | 5 | 2 | 5 | 12 |
| 1. Shared Facilities (School, Sport, Community, Recreation) | Structures | 1 | 4 | 9 | 14 | 5 | 9 | 7 | 21 | 4 | 7 | 8 | 19 |
| 1. Funding | Structures | 4 | 8 | 3 | 15 | 6 | 8 | 5 | 19 | 3 | 10 | 12 | 25 |
| 1. Inclusive/Diverse Facilities/Opportunities | Structures | 2 | 7 | 7 | 16 | 11 | 10 | 11 | 32 | 7 | 3 | 3 | 13 |
| 1. Self-Confidence, Capability and Competence | Events | 13 | 1 | 2 | 16 | 9 | 1 | 1 | 11 | 13 | 4 | 9 | 26 |
| 1. Recreation Infrastructure, Spaces and Places | Structures | 8 | 5 | 4 | 17 | 3 | 2 | 6 | 11 | 6 | 12 | 2 | 20 |
| 1. Local Social and Cultural Norms | Beliefs | 10 | 6 | 5 | 21 | 10 | 3 | 2 | 15 | 12 | 8 | 13 | 33 |
| 1. Community Resources, Training and Support | Structures | 5 | 9 | 8 | 22 | 8 | 6 | 8 | 22 | 2 | 5 | 7 | 14 |
| 1. Promotion/Marketing | Events | 7 | 10 | 10 | 27 | 12 | 12 | 12 | 36 | 1 | 1 | 1 | 3 |
| 1. Walking Culture | Beliefs | 6 | 12 | 12 | 30 | 1 | 5 | 9 | 15 | 9 | 11 | 4 | 24 |
| 1. Cycling Culture | Beliefs | 11 | 11 | 11 | 33 | 7 | 4 | 10 | 21 | 10 | 9 | 10 | 29 |
| 1. Crime And Anti-Social Behaviour | Goals | 12 | 13 | 13 | 38 | 13 | 13 | 13 | 39 | 11 | 13 | 11 | 35 |

*Notes:* ASM (Action Scales Model). G (group). Lower score indicates a greater rank
